# Dose–response relationship between serum *N*-glycan markers and liver fibrosis in chronic hepatitis B

**DOI:** 10.1007/s12072-024-10709-y

**Published:** 2024-07-17

**Authors:** Chi Zhang, Yiqi Liu, Lin Wang, Xueen Liu, Cuiying Chen, Junli Zhang, Chao Zhang, Guiqiang Wang, Hui Zhuang, Hong Zhao, China HepB-Related Fibrosis Assessment Research Group, Chi Zhang, Li-Hua Cao, Zhan-Qing Zhang, Wei-Feng Zhao, Qing-Hua Shang, Da-Zhi Zhang, An-Lin Ma, Qing Xie, Hong-Lian Gui, Guo Zhang, Ying-Xia Liu, Jia Shang, Shin-Bin Xie, Jun Li, Xu-Qing Zhang, Zhi-Qiang Zou, Yu-Ping Chen, Zong Zhang, Ming-Xiang Zhang, Jun Cheng, Fu-Chun Zhang, Li-Hua Huang, Jia-Bin Li, Qing-Hua Meng, Hai-Bin Yu, Yu-Qiang Mi, Yan-Zhong Peng, Zhi-Jin Wang, Li-Ming Chen, Fan-Ping Meng, Wan-Hua Ren, Lang Bai, Yi-Lan Zeng, Rong Fan, Xian-Zhi Lou, Wei-Feng Liang

**Affiliations:** 1 Department of Infectious Disease, Center for Liver Disease, Peking University First Hospital, Beijing, 100034 China; 2 Department of Clinical Laboratory, Beijing Chao-Yang Hospital, Capital Medical University, Beijing, 100020 China; 3 Department of Microbiology & Center of Infectious Diseases, School of Basic Medical Sciences, Peking University Health Science Center, Beijing, 100191 China; 4 Department of Research and Development, Sysdiagno (Nanjing) Biotech Co., Ltd, Nanjing, 210008 Jiangsu Province China; 5 Department of Infectious Diseases, Peking University International Hospital, Beijing, 102206 China; 6 Beijing Key Laboratory of Hepatitis C and Immunotherapy for Liver Diseases, Beijing, China

**Keywords:** Chronic hepatitis B, Liver fibrosis, Serum *N*-glycan markers, Diagnostic performance, RNA-transcriptome sequencing

## Abstract

**Background:**

Evaluation of liver fibrosis played a monumental role in the diagnosis and monitoring of chronic hepatitis B (CHB). We aimed to explore the value of serum* N*-glycan markers in liver fibrosis.

**Methods:**

This multi-center (33 hospitals) study recruited 760 treatment-naïve CHB patients who underwent liver biopsy. Serum *N*-glycan markers were analyzed by DNA sequencer-assisted fluorophore-assisted with capillary electrophoresis (DSA-FACE) technology. First, we explore the relationship between 12 serum *N*-glycan markers and the fibrosis stage. Then, we developed a Px score for diagnosing significant fibrosis using the LASSO regression. Next, we compared the diagnostic performances between Px, LSM, APRI, and FIB-4. Finally, we explored the relationships between glycosyltransferase gene and liver fibrosis with RNA-transcriptome sequencing.

**Results:**

We included 622 CHB participants: male-dominated (69.6%); median age 42.0 (IQR 34.0–50.0); 287 with normal ALT; 73.0% with significant fibrosis. P5(NA2), P8(NA3), and P10(NA4) were opposite to the degree of fibrosis, while other profiles (except for P0[NGA2]) increased with the degree of fibrosis. Seven profiles (P1[NGA2F], P2[NGA2FB], P3[NG1A2F], P4[NG1A2F], P7[NA2FB], P8[NA3], and P9[NA3Fb]) were selected into Px score. Px score was associated with an increased risk of significant fibrosis (for per Px score increase, the risk of significant fibrosis was increased by 3.54 times (OR = 4.54 [2.63–7.82]) in the fully-adjusted generalized linear model. *p* for trend was <0.001. The diagnostic performance of the Px score was superior to others. Glycosyltransferase genes were overexpressed in liver fibrosis, and glycosylation and glycosyltransferase-related pathways were significantly enriched.

**Conclusions:**

Serum *N*-glycan markers were positively correlated with liver fibrosis. Px score had good performance in distinguishing significant fibrosis.

**Supplementary Information:**

The online version contains supplementary material available at 10.1007/s12072-024-10709-y.

## Introduction

As of 2022, there were 257.5 million (216.6–316.4) individuals positive for HBsAg globally [1]. Decompensate cirrhosis and hepatocellular carcinoma (HCC) were the main causes of death in chronic HBV-infected individuals [2–4]. Over 85% of HCC was caused by HBV infection (only HBV positive 83.77%; HBV + HCV positive 1.64%) in China [5, 6]. Timely antiviral treatment can alleviate the progression of liver fibrosis, and even reverse significant liver fibrosis/cirrhosis, thereby reducing the occurrence of decompensated cirrhosis and HCC. Marcellin et al. study with 348 paired liver biopsies at baseline and 240 weeks indicated that: 51% (176/348) of patients had regression of fibrosis at week 240; 74% (71/96) of patients with baseline cirrhosis achieved cirrhosis reversal; only 3 of 252 patients without cirrhosis at baseline progressed to cirrhosis at year 5 [7].

Accurately evaluating the fibrosis stages were of great value in the diagnosis and monitoring of chronic hepatitis B (CHB). The methods of evaluating fibrosis included liver biopsy and noninvasive methods [8]. Although liver biopsy was the standard in diagnosing fibrosis stages, its undeniable complications, including pain (in 30–50% of patients), serious bleeding (0.6%), injury to other organs (0.08%), and in rare cases death (up to 0.1%) [8]. For these reasons, many patients even physicians are reluctant to undergo liver biopsy [9]. Noninvasive methods (including liver stiffness measurements [LSM, elastography] and serological markers) may be used instead of liver biopsies to assess for the severity of fibrosis [3]. What’s more, noninvasive fibrosis evaluation indicators were closely related to the prognosis of patients. An international multicenter study by Serra-Burriel et al. [10] (included 416,200 participants) demonstrated that compared to the low-risk group, the high-risk group had a 470-fold increase in liver-related mortality (HR 471 [95% CI 347–641]). Fibrosis-4 index (FIB-4) or aspartate aminotransferase (AST) to platelet ratio index (APRI) scores also had similar values [10].

Glycosylation was one of the important post-translational modifications of proteins [11]. It was estimated that approximately 50% of human proteins were glycoproteins, and most of them contain *N*‑glycan structures [12]. Serum *N*-glycan had considerable value in the evaluation of liver diseases. Our previous study (with 450 CHB patients) indicated that branch alpha (1,3)-fucosylated triantennary glycan was more abundant in patients with HCC than cirrhosis (median 3.7 [95% CI 3.5–3.9] vs. 2.3 [2.0–2.6]); *N*-glycan markers were also superior to AFP in diagnosing HCC (AUROC 0.81 vs. 0.78) [13]. Several studies (10 citations) also supported glycomics as diagnostic markers for HCC [14]. In addition, our study also found that *N*-glycan markers using machine-learning approaches could effectively diagnose significant fibrosis and cirrhosis in CHB patients with normal ALT levels [15].

Based on our previous research, we will comprehensively analyze the diagnostic value of serum *N*-glycan markers for significant fibrosis in chronic HBV-infected individuals and preliminarily explore the relationship between the expression of glycosylation-related genes and fibrosis.

## Methods

### Participants

All participants in this study were from a randomized controlled study (NCT03568578), and these participants came from 33 hospitals in Chinese Mainland (Figure S1). The inclusion and exclusion criteria have been described in previous studies [16]. All enrolled patients were treatment-naïve CHB patients (HBsAg positive >6 months) and with liver biopsy results. Exclusion criteria included co-infection with other hepatitis viruses (hepatitis C virus [HCV], hepatitis D virus [HDV]) or co-infection with human immunodeficiency virus (HIV). Other chronic liver diseases, including autoimmune hepatitis, drug-induced liver injury (DILI), genetic, and nonalcoholic fatty liver disease (NAFLD) were also excluded. Due to the serum *N*-glycan markers might be affected by hepatocellular carcinoma (HCC), all suspected HCC individuals were excluded. The detailed enrollment strategies are shown in Figure S2.

This study was approved by the Ethical Committees of Peking University First Hospital and participating hospitals. All patients signed informed consent before enrollment. This study was done in accordance with the principles of the Declaration of Helsinki and the International Conference on Harmonization-Good Clinical Practice guidelines.

### Data acquisition and laboratory evaluation

Information on demographic (age, sex, body mass index [BMI], family history of CHB or HCC) and clinical data (white blood cell [WBC], hemoglobin [HGB], platelet [PLT], alanine aminotransferase [ALT], aspartate aminotransferase [AST], alkaline phosphatase [ALP], glutamyl transpeptidase [GGT], albumin [ALB], total bilirubin [TBIL], direct bilirubin [DBIL], total glyceride [TG], total cholesterol [TC], high-density lipoprotein [HDL], low-density lipoprotein [LDL], alpha-fetoprotein [AFP], prothrombin activity [PTA]) were available from each participant center. All data were collected within 2 weeks before liver biopsy. Virological markers (HBV DNA, HBsAg, HBeAg, Anti-HBe, and qAnti-HBc) were tested in the central laboratory uniformly.

Serum HBV DNA was quantified using Roche kits (COBAS AmpliPrep/COBAS TaqMan), with a detection range of 20 IU/mL to 1.7 × 10^8^ IU/mL. The samples were tested at dilutions of 1:10 to 1:100,000 (tenfold increase) if the HBV DNA level was >1.7 × 10^8^ IU/mL. HBsAg, HBeAg, and anti-HBe were tested using enzyme immunoassay kits (Roche Diagnostics, Penzberg, Germany) according to the instructions. The serum qAnti-HBc level was measured using a newly developed chemiluminescent microparticle immunoassay (dynamic range 100–100000 IU/mL; Wantai, China). AST-to-platelet ratio index (APRI) was calculated as APRI = [AST/AST (ULN) × 100]/PLT (×10^9^/L); fibrosis index based on four factors (FIB-4) was calculated as FIB-4 = age × AST/[PLT (×10^9^/L) × √ALT].

### Serum *N*-glycan detection

**Fig. 1:**
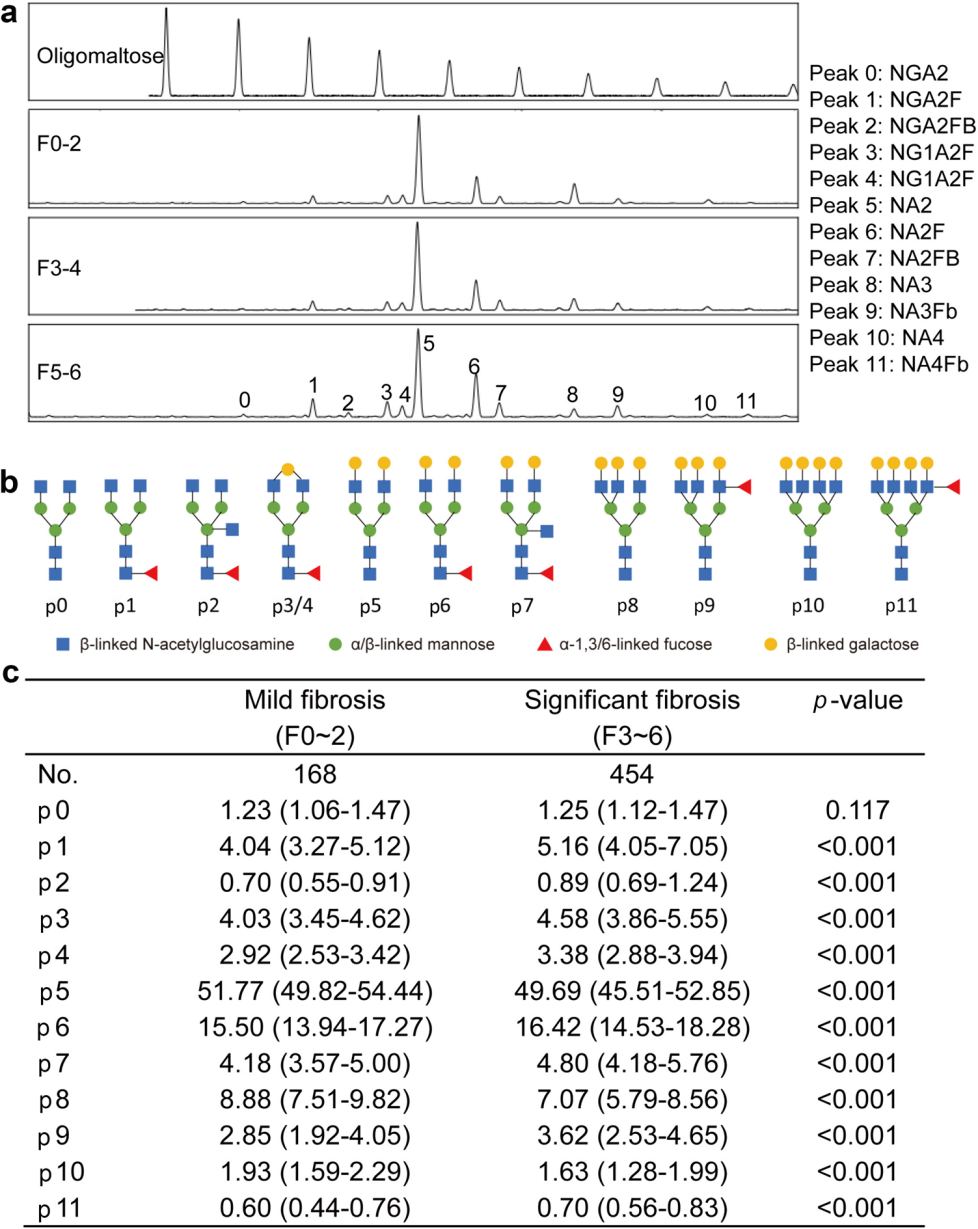
The value of 12 *N*-glycan peaks in different liver fibrosis stage. **a** Representative *N*-glycan peaks of different liver fibrosis stages; **b** structure of 12 *N*-glycan peaks; **c** *N*-glycan profiles abundances between different liver fibrosis stages. Fibrosis stages were evaluated by Ishak scoring system, and significant fibrosis was defined as *F* ≥ 3. The data in figure C was represented as the median (interquartile range), and student *t*-test (Gaussian distribution) or Kruskal–Wallis *H*-test (skewed distribution) were used to detect the differences among fibrosis stage

The *N*-glycan present on the protein in 2 μL of serum were released, labeled, and analyzed as described previously [13, 15]. Serum glycoprotein *N*-glycome profiling was performed following the instructions of the Glycan-Test Kit (Sysdiagno Biomedtech, Jiangsu, China). Labeled *N*-glycans were analyzed by DNA sequencer-assisted fluorophore-assisted with capillary electrophoresis (DSA-FACE) technology with a capillary electrophoresis-based ABI 3500 Dx sequencer (Applied Biosystems, USA). Serum *N*-glycan profile data were analyzed using GeneMapper software version 4.1 (Applied Biosystems, USA). Twelve specific serum *N*-glycan peaks were obtained in each sample (Fig. 1), and the abundance of each peak was quantified by normalizing its height to the sum of the heights of twelve peaks.

### Liver histological examination

After signing informed consent, all patients underwent ultrasonographic-guided liver biopsy according to standard procedures in each hospital. The specimens were fixed with 10% neutral formalin and then embedded in paraffin. All specimens underwent hematoxylin–eosin (HE), Masson’s trichrome, and reticular fiber staining. Liver biopsy samples with a length exceeding 15 mm and more than 6 portal areas were evaluated as qualified samples. The necroinflammation grading and fibrosis staging scores of the same sample were determined by two pathologists from Youan Hospital Capital Medical University, blindly. If the difference in necroinflammation scores exceeds 2 points, or the fibrosis score exceeds 1 point between two individuals, the sample was viewed by a third experienced pathologist and they negotiated and gave final scores.

Liver necroinflammation (modified histology activity index, HAI) grade and fibrosis stage were evaluated by Ishak scoring system [17], with necroinflammation scores ranging from 0 to 18 and fibrosis scores from 0 to 6. Significant fibrosis was defined as *F* ≥ 3, and cirrhosis was defined as *F* ≥ 5.

### Bioinformatics analysis of glycosyltransferase and liver fibrosis

Microarray datasets were screened from GEO. The search keywords were “hepatitis B” and “liver biopsy” with sample size exceeding 50 cases. Finally, GSE84044 was selected, which contained 124 cases (mild fibrosis 63; significant fibrosis 61).

We transformed the probe into a gene symbol based on the platform’s annotation file (GPL570), when there were multiple probes mapped to the same gene symbol; the maximum value of probes was selected as the gene expression value. Differentially expressed genes (DEGs) between mild and significant fibrosis were analyzed via the “limma package” in R software, with the cutoff: *p* < 0.05 and fold changes (FC) >1.2. Collection and collation of glycosylation-related genes from the “GSEA” database (GOBP_GLYCOSYLATION). The intersection of DEGs and glycosylation-related genes was visualized by the Venn plot.

We used the “clusterProfiler” package of R to perform the Gene Ontology (GO) and Kyoto Encyclopedia of Genes and Genomes (KEGG) enrichment analyses of glycosylation-related DEGs. GO analysis included three categories, biological process (BP), cellular component (CC), and molecular function (MF), which was important in the exploration of biological functions. KEGG analysis was used to explore potential pathways.

### Statistical analysis

Before inferential analyses, HBV DNA, HBsAg, and qAnti-HBc were log_10_ transformation. Continuous variables were described as mean (standard deviation, SD) and median (interquartile range, IQR), and student’s *t*-test (Gaussian distribution) or Kruskal–Wallis *H*-test (skewed distribution) were used to detect the differences among fibrosis stage (binary variable). Categorical variables were described as frequency (percentage), and the difference between them was compared with Chi-square or Fisher’s exact tests. This study was in accordance with the Strengthening Reporting of Observational Studies in Epidemiology (STROBE) statement [18].

Our statistical analyses consisted of three main steps. In Step 1, we analyzed the differences in demographic, laboratory tests, and serum *N*-glycan markers (Peak 0 to Peak 11) between two groups (*F* 0–2 vs. 3–6) on fibrosis staging. Then, comparing the differences among them in three groups (*F* 0–2 vs. 3–4 vs. 5–6). In Step 2, to more accurately assess the relationship between *N*-glycan markers and significant fibrosis risk, we employed restricted cubic spline (RCS) to explore the correlation between them. The number of nodes were four in each peak. Because the position of nodes had a mild effect on the fitting of RCS, the positions of nodes were automatically selected.

In Step 3, based on the significantly different *N*-glycan markers, we developed a model Px for diagnosing significant fibrosis using least absolute shrinkage and selection operator (LASSO) regression. To examine the correlation between Px and risk of significant fibrosis, we constructed three distinct models using linear regression models based on the generalized linear model (GLM), including non-adjusted model (no covariates were adjusted), adjusted I model (sex, age, and BMI were adjusted) and adjust II (adjust for age, sex, BMI, PLT, ALT, AST, ALP, GGT, ALB, and LSM were adjusted). Effect sizes (odds ratio) with 95% CI were recorded. Next, nonlinearity between Px and fibrosis was addressed using the aforementioned RCS regression. To test the robustness of our results, we performed subgroup analysis and sensitivity analysis. We identified the relationship between the whole participants and the ALT normal participants, separately. For Px, we converted it into a categorical variable according to the tertile and calculated the *p* for trend to verify the results of Px as the continuous variable.

In Step 4, we compared the diagnostic value between Px, LSM, APRI, and FIB-4. All non-invasive models were used to fit the receiver operating characteristic (ROC) curves for diagnosing significant fibrosis whole participants and the ALT normal participants. Then, we calculated the area under ROC (AUROC), sensitivity, specificity, positive predictive value (PPV), and negative predictive (NPV) value.

All analyses were performed with R software (http://www.Rproject.org, The R Foundation) and EmpowerStats (http://www.empowerstats.com, X&Y Solutions, Inc). *p* values less than 0.05 (two-sided) were considered statistically significant.

## Results

### Baseline characteristics of participants

We recruited 760 CHB participants from 2018 to 2020 in 33 hospitals. Finally, 622 CHB patients (287 with normal ALT) were enrolled in our study (Figure S1, and Figure S2). As shown in Table 1, the proportion of males (72.0 vs. 63.1%, *p* = 0.032), mean age (42.7 ± 10.2 vs. 40.7 ± 9.9, *p* = 0.044) and BMI (24.1 ± 3.5 vs. 23.3 ± 3.0, *p* = 0.008) were slightly higher in significant fibrosis group. In biochemical and blood tests, significant fibrosis patients had higher levels of ALT (median 45.0 vs. 31.5, *p* < 0.001), AST (median 36.0 vs. 27.8, *p* < 0.001), ALP (median 82.0 vs. 76.0, *p* < 0.001), and GGT (median 38.0 vs. 22.0, *p* < 0.001), while PLT (median 160 vs. 192, *p* < 0.001) and ALB (median 42.3 vs. 45.0, *p* < 0.001) were opposite. In virology, HBV DNA titers and proportion of HBeAg positivity increased with the fibrosis stage, while there was no significant difference in HBsAg and qAnti-HBc levels. As expected, the population with significant fibrosis had higher non-invasive fibrosis markers (APRI, FIB-4, LSM, all *p* < 0.001), and necroinflammation scores (*p* < 0.001) were higher in significant fibrosis stage patients. Table S1 shows the differences in fibrosis stage (*F* 0–2 vs. 3–4 vs. 5–6).

**Table 1:** Baseline characteristics of all participants

	Mild fibrosis (*F* 0~2)	Significant fibrosis (*F* 3~6)	*p *value
No	168	454	
Age (year)	40.7 (9.9) 41.0 (33.8–47.0)	42.7 (10.2) 42.0 (34.0–50.0)	0.044
BMI (kg/m^2^)	23.3 (3.0) 23.0 (21.2–25.1)	24.1 (3.5) 23.9 (21.7–26.2)	0.008
Sex			0.032
Female	62 (36.9%)	127 (28.0%)	
Male	106 (63.1%)	327 (72.0%)	
WBC (×10^^9^)	5.4 (1.3) 5.2 (4.5–6.2)	5.4 (1.6) 5.2 (4.3–6.2)	0.743
HGB (g/L)	144.5 (16.9) 145.0 (134.8–155.2)	145.5 (17.0) 148.0 (136.0–159.0)	0.153
PLT (×10^^9^)	194.8 (54.2) 192.0 (162.0–223.2)	163.4 (52.3) 160.0 (124.0–196.0)	<0.001
ALT (U/L)	54.8 (73.2) 31.5 (23.6–50.5)	95.9 (162.1) 45.0 (31.0–83.8)	<0.001
AST (U/L)	37.4 (36.0) 27.8 (21.0–37.0)	71.0 (136.8) 36.0 (27.0–57.0)	<0.001
ALP (U/L)	75.8 (20.6) 76.0 (63.8–87.0)	88.2 (34.5) 82.0 (65.2–103.8)	<0.001
GGT (U/L)	33.0 (32.6) 22.0 (15.8–38.0)	62.1 (73.3) 38.0 (24.0–76.0)	<0.001
ALB (g/L)	44.8 (3.9) 45.0 (42.6–47.0)	42.3 (4.6) 42.3 (39.1–45.8)	<0.001
TBIL (μmol/L)	15.1 (6.5) 13.7 (10.7–18.4)	17.9 (14.4) 15.2 (11.6–20.5)	0.006
DBIL (μmol/L)	4.2 (2.5) 3.7 (2.7–5.3)	6.1 (8.0) 4.6 (3.4–6.7)	<0.001
TCHO (mmol/L)	4.6 (0.9) 4.6 (4.1–5.2)	4.4 (0.9) 4.3 (3.8–4.9)	<0.001
TG (mmol/L)	1.2 (0.6) 1.0 (0.8–1.4)	1.2 (0.7) 1.0 (0.8–1.4)	0.573
HDL (mmol/L)	1.4 (0.4) 1.4 (1.1–1.6)	1.3 (0.3) 1.3 (1.1–1.5)	0.004
LDL (mmol/L)	2.8 (0.7) 2.7 (2.2–3.2)	2.6 (0.8) 2.5 (2.0–3.0)	0.003
AFP (ng/mL)	8.2 (32.9) 2.9 (1.9–4.5)	17.6 (50.2) 4.5 (2.5–10.5)	<0.001
PTA (%)	99.2 (12.2) 100.0 (93.0–102.9)	89.5 (12.9) 90.5 (81.0–100.0)	<0.001
LSM (kPa)	7.2 (3.7) 6.1 (4.8–8.8)	14.2 (9.5) 11.8 (7.9–17.3)	<0.001
CAP (dB/m)	222.7 (47.0) 219.0 (198.0–249.0)	218.1 (49.2) 217.5 (188.8–249.0)	0.437
APRI	0.5 (0.6) 0.4 (0.3–0.5)	1.3 (3.0) 0.6 (0.4–1.1)	<0.001
FIB-4	1.2 (0.7) 1.0 (0.8–1.5)	2.0 (2.0) 1.6 (1.0–2.3)	<0.001
HBV DNA (lg IU/mL)	5.0 (2.3) 4.6 (3.3–7.1)	5.4 (1.9) 5.4 (3.9–6.8)	0.027
HBsAg (lg IU/mL)	3.2 (1.0) 3.3 (2.7–3.8)	3.3 (0.8) 3.3 (3.0–3.6)	0.601
HBeAg			0.001
Negative	112 (67.1%)	239 (52.8%)	
Positive	55 (32.9%)	214 (47.2%)	
HBeAb			0.981
Negative	53 (31.7%)	143 (31.6%)	
Positive	114 (68.3%)	309 (68.4%)	
qAnti-HBc (lg IU/mL)	3.9 (0.9) 4.1 (3.5–4.5)	4.0 (0.7) 4.1 (3.5–4.5)	0.689
HBV family history			0.089
No	83 (49.4%)	259 (57.0%)	
Yes	85 (50.6%)	195 (43.0%)	
HCC family history			0.048
No	140 (83.3%)	405 (89.2%)	
Yes	28 (16.7%)	49 (10.8%)	
HAI (Ishak)	3.7 (2.2) 3.0 (2.0–4.0)	6.0 (2.8) 6.0 (4.0–7.0)	<0.001
0~4	130 (77.4%)	151 (33.3%)	
5~6	23 (13.7%)	144 (31.7%)	
7~9	11 (6.5%)	108 (23.8%)	
10~18	4 (2.4%)	51 (11.2%)	
Splenomegaly			<0.001
No	126 (88.7%)	277 (68.4%)	
yes	16 (11.3%)	128 (31.6%)	

Data presented as mean (standard deviation) and median (quartile), while Gaussian distribution, compared with Student’s *t*-test; Skewed distribution, compared with Kruskal–Wallis analysis) for continuous variables; number (percentage) for categorical variables (Chi-square or Fisher’s exact tests). Fibrosis was measured by Ishak scoring system

*BMI* body mass index, *WBC* white blood cell, *HGB* hemoglobin, *PLT* platelet, *ALT* alanine aminotransferase, *AST* aspartate aminotransferase, *ALP* alkaline phosphatase, *GGT* glutamyl transpeptidase, *ALB* albumin, *TBIL* total bilirubin, *DBIL* direct bilirubin, *TG* total glyceride, *TC* total cholesterol, *HDL* high density lipoprotein, *LDL* low density lipoprotein, *AFP* alpha fetoprotein, *PTA* prothrombin activity, *HAI* histology activity index by Ishak fibrosis score, *LSM* liver stiffness measurement, *APRI*, AST-to-platelet ratio index, [(AST/ULN) × 100/PLT], *FIB-4* fibrosis index based on four factors, {(age × AST)/[PLT × (ALT^0.5)]}

### Serum *N*-glycan peaks in different stages of liver fibrosis

We used DSA-FACE technology to detect serum *N*-glycan profiles and identified 12 *N*-glycan peaks (P0-P11) in each subject. Figure 1A showed representative *N*-glycan profiles of different degrees of fibrosis. The patterns of various *N*-glycan peaks were shown in Fig. 1B. Except for P0, P5, P8, and P10, all others contain α-1,3/6-linked fucose. The sum of *N*-glycan profile values for each subject was 100. We found that significant differences (all *p* < 0.001) in *N*-glycan profiles except for P0 (NGA2). P5 (NA2), P8 (NA3), and P10 (NA4) were opposite to the degree of fibrosis, while other profiles increased with the degree of fibrosis (Fig. 1C).

**Fig. 2:**
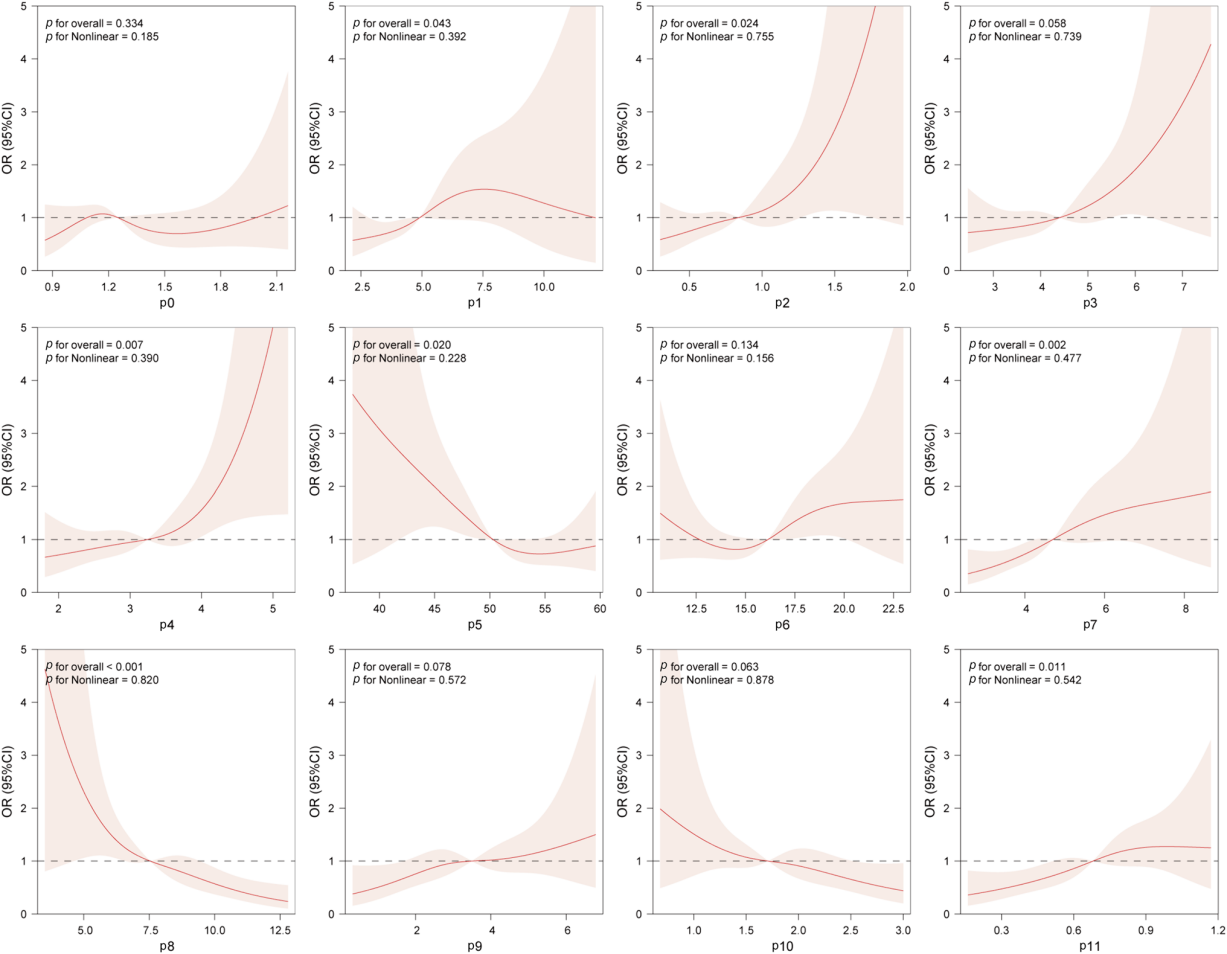
Correlation between 12 *N*-glycan peaks abundances and risk of significant fibrosis by restricted cubic spline

Subsequently, we used the RCS regression model to explore the correlation between each *N*-glycan profile and significant fibrosis, as well as whether there were non-linear relationships (Fig. 2, Table S2). Our results indicated that P1 (NGA2F), P2 (NGA2FB), P4 (NG1A2F), P5 (NA2), P7 (NA2FB), P8 (NA3), and P11 (NA4Fb) were significantly associated with the risk of fibrosis (all *p* < 0.05), while P5 (NA2) and P8 (NA3) were negatively correlated with profiles. No non-linear relationship was found among all profiles (all *p* > 0.05).

### Development of serum *N*-glycan models Px

**Fig. 3:**
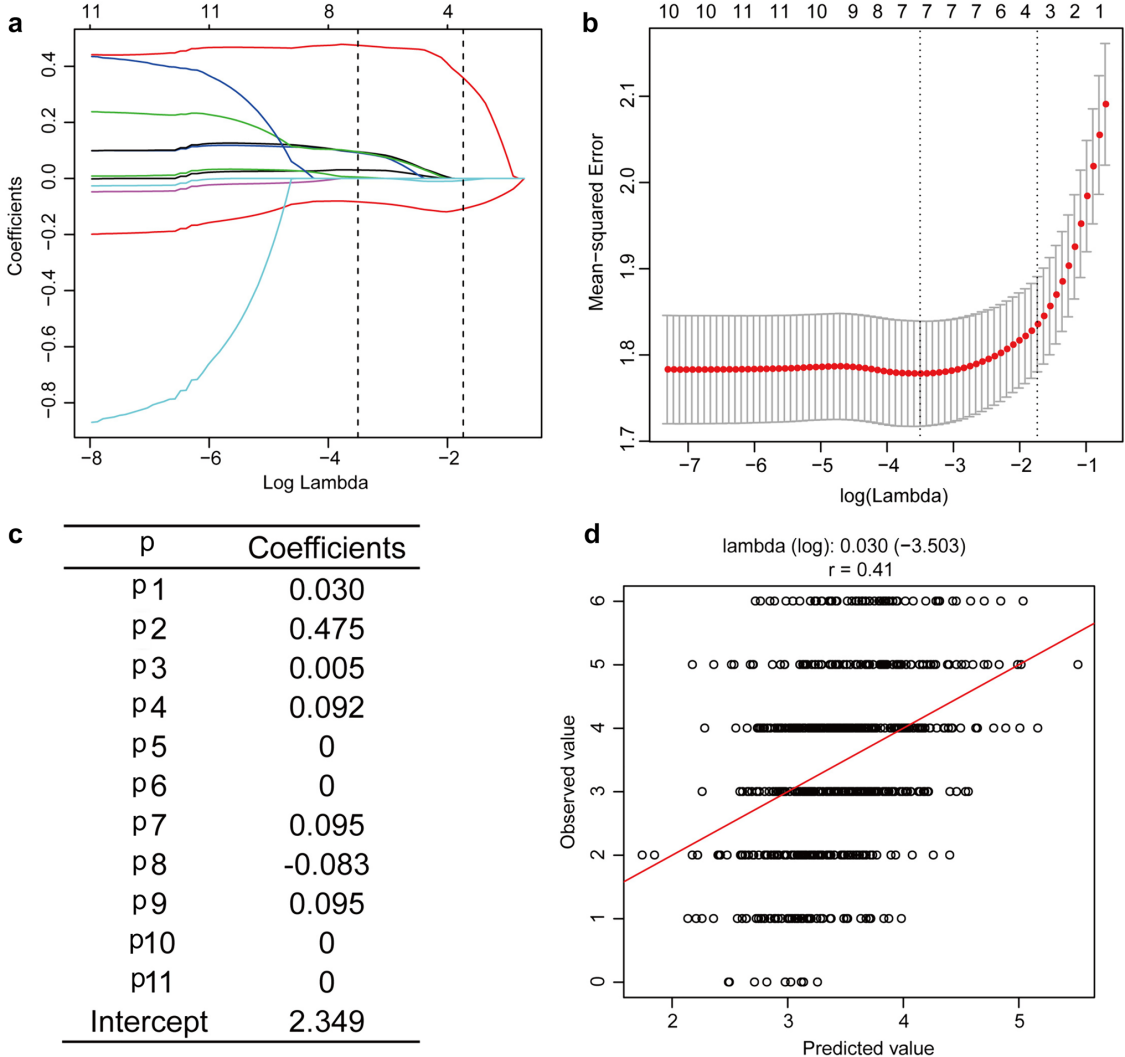
Developing a significant fibrosis prediction model Px score using least absolute shrinkage and selection operator (LASSO) regression. **a** LASSO coefficient path; **b** LASSO regularization path; **c** coefficients of each *N*-glycan peak; **d** correlation between predicted value and observed value

Based on the differential *N*-glycan profiles, we constructed a model Px by LASSO regression to diagnosing significant fibrosis. Ultimately, 7 profiles (P1 [NGA2F], P2 [NGA2FB], P3 [NG1A2F], P4 [NG1A2F], P7 [NA2FB], P8 [NA3], and P9 [NA3Fb]) were selected into Px model. The formula of telling apart significant fibrosis was: Px = 0.030 × P1 + 0.475 × P2 + 0.005 × P3 + 0.092 × P4 + 0.095 × P7-0.083 × P8 + 0.095 × P9 + 2.349, with a correlation coefficient 0.43 (Fig. 3).

**Fig. 4:**
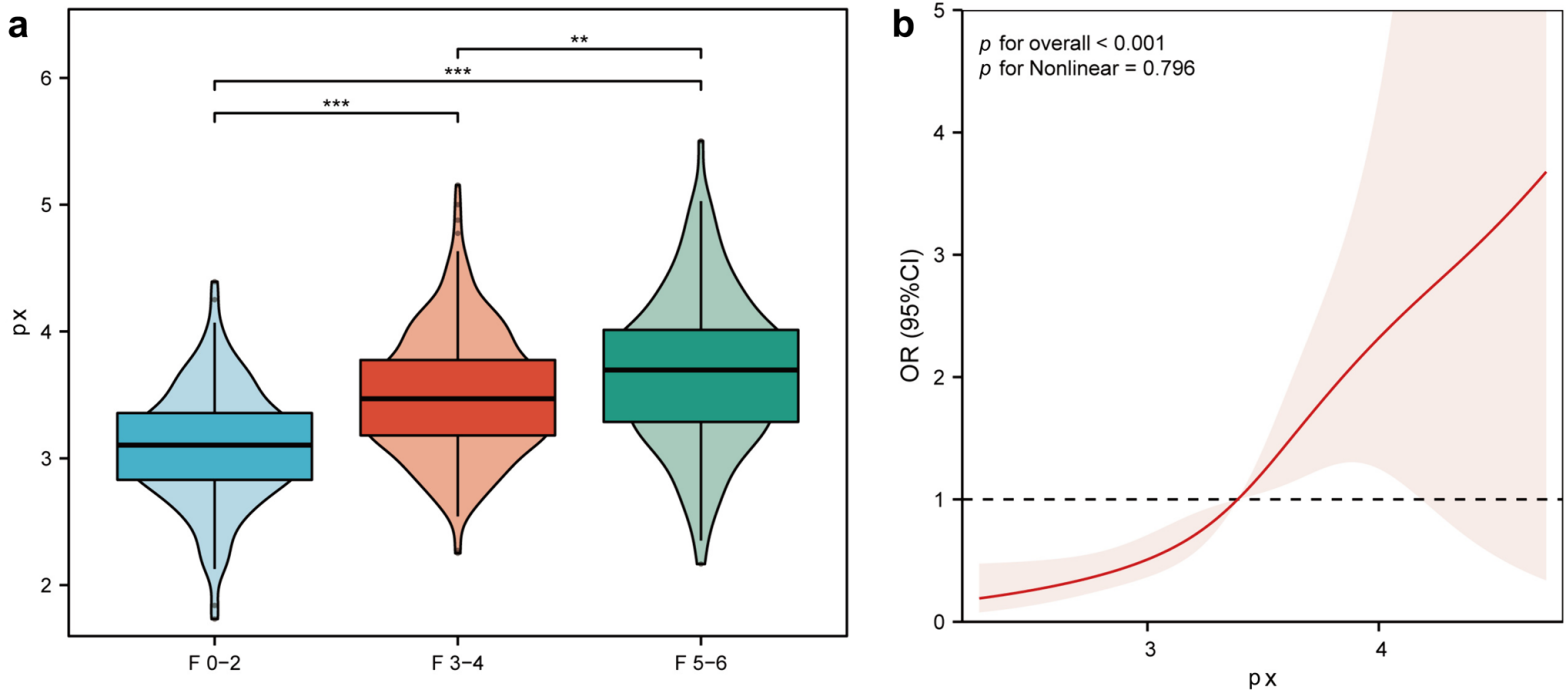
The diagnostic value of Px for significant liver fibrosis. **a** Distribution of Px among different fibrosis stage **b** restricted cubic spline of Px for diagnosis of significant fibrosis

Px was positively correlated with the degree of fibrosis, with median values of 3.10 (2.83–3.36), 3.47 (3.18–3.78), and 3.696 (3.29–4.01) for fibrosis stage F0–2, F3–4, and F5–6, respectively (Fig. 4). The RCS results were also consistent (*p* < 0.001).

### Relationship between Px score and risk of significant fibrosis

We used GLM to analyze the relationship between Px and liver fibrosis, as shown in Table 2. In all participants, in the non-adjusted model, the Px score was associated with an increased risk of significant fibrosis (for per Px score increase, the risk of significant fibrosis was increased by 7.29 times, (OR = 8.29 [5.21, 13.20]). In the adjusted I models (adjust for age, sex, and BMI; OR = 8.98 [5.48, 14.74]), and adjusted II models (adjust for age, sex, BMI, PLT, ALT, AST, ALP, GGT, ALB, and LSM; OR = 4.54 [2.63, 7.82]) were consistent (all *p* < 0.001). In the adjusted II model, compared with the Px score bottom tertile, patients in the middle tertile (OR = 2.53, [1.57, 4.08]) and top tertile (OR = 4.58, [2.41, 8.70]) had an increased risk of significant fibrosis. For sensitivity analysis, we also handled the Px score as a categorical variable (tertile) and found the same trend (*p* for trend <0.001).

**Table 2:** Relationship between Px and fibrosis in all and ALT normal participants

Variable	Non-adjusted		Adjust I		Adjust II	
	OR (95% CI)	*p* value	OR (95% CI)	*p* value	OR (95% CI)	*p* value
All participates^1^						
Px score increase	8.29 (5.21, 13.20)	<0.001	8.98 (5.48, 14.74)	<0.001	4.54 (2.63, 7.82)	<0.001
Px (tertile)						
Bottom tertile (1.73–3.18)	Reference		Reference		Reference	
Middle tertile (3.18–3.64)	3.28 (2.15, 5.01)	<0.001	3.36 (2.17, 5.21)	<0.001	2.53 (1.57, 4.08)	0.001
Top tertile (3.64–5.50)	11.13 (6.32, 19.60)	<0.001	11.68 (6.46, 21.10)	<0.001	4.58 (2.41, 8.70)	<0.001
*p* for trend	–	<0.001	–	<0.001	–	<0.001
ALT normal participates^2^						
Px score increase	10.41 (5.05, 21.47)	<0.001	10.48 (4.92, 22.33)	<0.001	6.53 (2.81, 15.18)	<0.001
Px (tertile)						
Bottom tertile (1.73–3.18)	Reference		Reference		Reference	
Middle tertile (3.18–3.63)	3.72 (2.09, 6.63)	<0.001	3.67 (2.03, 6.65)	<0.001	3.00 (1.56, 5.77)	0.001
Top tertile (3.64–5.01)	7.07 (3.22, 15.55)	<0.001	6.98 (3.09, 15.76)	<0.001	3.01 (1.21, 7.48)	0.018
*p* for trend	–	<0.001	–	<0.001	–	0.002

Non-adjusted model: adjust for None

Adjust I model: adjust for age, sex, and BMI

Adjust II model: adjust for age, sex, BMI, PLT, ALT, AST, ALP, GGT, ALB, and LSM

^1^Number of cases in “all participates” in non-adjusted, adjust I and adjust II were 622, 622 and 618

^2^Number of cases in “ALT normal participates” in non-adjusted, adjust I and adjust II were 287, 287 and 286

In the ALT normal participants, both non-adjusted, adjusted I, and adjusted II models showed a significant positive correlation between Px score and fibrosis (Table 2; all *p* < 0.001). In the adjusted II model, for per score of Px increase, the risk of significant fibrosis increases by 5.53 times (OR = 6.53 [2.81, 15.18]). Compared to the lower tertile, patients in the middle and top tertile showed a significant twofold and 2.01-fold increase in fibrosis risk, respectively. The trend test of positive correlation between Px score and fibrosis was significant (*p* = 0.002). In addition, Px score still had good diagnostic value in the population with elevated ALT (not shown).

### Efficacy of Px in diagnosing significant fibrosis

We compared the Px score with other non-invasive indicators (LSM, APRI, and FIB-4) for diagnosing liver fibrosis (Table 3). The AUROC of Px score was 0.760 (0.719–0.801), which was higher than LSM (0.714 [0.673–0.755]), APRI (0.704 [0.658–0.749]), and FIB-4 (0.682 [0.637–0.727]). The sensitivity and specificity of Px in diagnosing significant fibrosis were 0.621 and 0.792, respectively. Especially the positive predictive value (ie. probability of significant fibrosis with a positive diagnosis of Px), the Px score was significantly better than other indicators (0.890). To further validate the diagnostic efficacy of Px, we randomly divided the study population into a training set and a validation set in a 1:1 ratio. As shown in Table S4, there were no significant differences in demographic, virological, and biochemical tests between the two groups (all *p* > 0.05). In addition, there were also no significant difference in the distribution of *N*-glycan profiles (Table S5; all *p* > 0.05). In the training set, the AUROC of Px score of diagnosis significant fibrosis was 0.754 (0.694–0.814), which was not inferior to LSM (0.756), APRI (0.743), and FIB-4 (0.712). In the validation set, the AUROC of Px score (0.766) was significantly higher than that of LSM, APRI, and FIB-4 (Table S6).

**Table 3:** Comparison of the efficacy of serum *N*-glycan Px model, LSM, APRI, and FIB-4 in diagnosing significant fibrosis

	AUROC (95% CI)	Sensitivity	Specificity	PPV	NPV	PLR	NLR
All participates							
Px	0.760 (0.719–0.801)	0.621	0.792	0.890	0.436	2.982	0.479
LSM	0.714 (0.673–0.755)	0.797	0.631	0.854	0.535	2.161	0.321
APRI	0.704 (0.658–0.749)	0.604	0.750	0.867	0.412	2.414	0.529
FIB-4	0.682 (0.637–0.727)	0.460	0.816	0.871	0.359	2.495	0.662
ALT normal							
Px	0.747 (0.689–0.805)	0.683	0.702	0.801	0.557	2.292	0.452
LSM	0.677 (0.621–0.733)	0.661	0.692	0.791	0.537	2.149	0.489
APRI	0.683 (0.621–0.745)	0.525	0.760	0.793	0.476	2.182	0.626
FIB-4	0.642 (0.577–0.707)	0.361	0.856	0.815	0.432	2.501	0.747

In the ALT normal population, we further compared their diagnostic efficacy. The AUROC was also the highest (0.747 vs. 0.677 vs. 0.683 vs. 0.642). These results indicated that the serum *N*-glycan Px score can effectively help us identify significant fibrosis.

### Relationship between glycosyltransferases and liver fibrosis

To further elucidate serum *N*-glycan and liver fibrosis, we explored the relationship between them with bioinformatics analysis. Firstly, we retrieved CHB patients with liver biopsy from the GEO database. A total of 124 patients (mild fibrosis 63; significant fibrosis 61) were included (GSE84044), with a median age of 40 (33–51) years, and 88 (71%) were male.

**Fig. 5:**
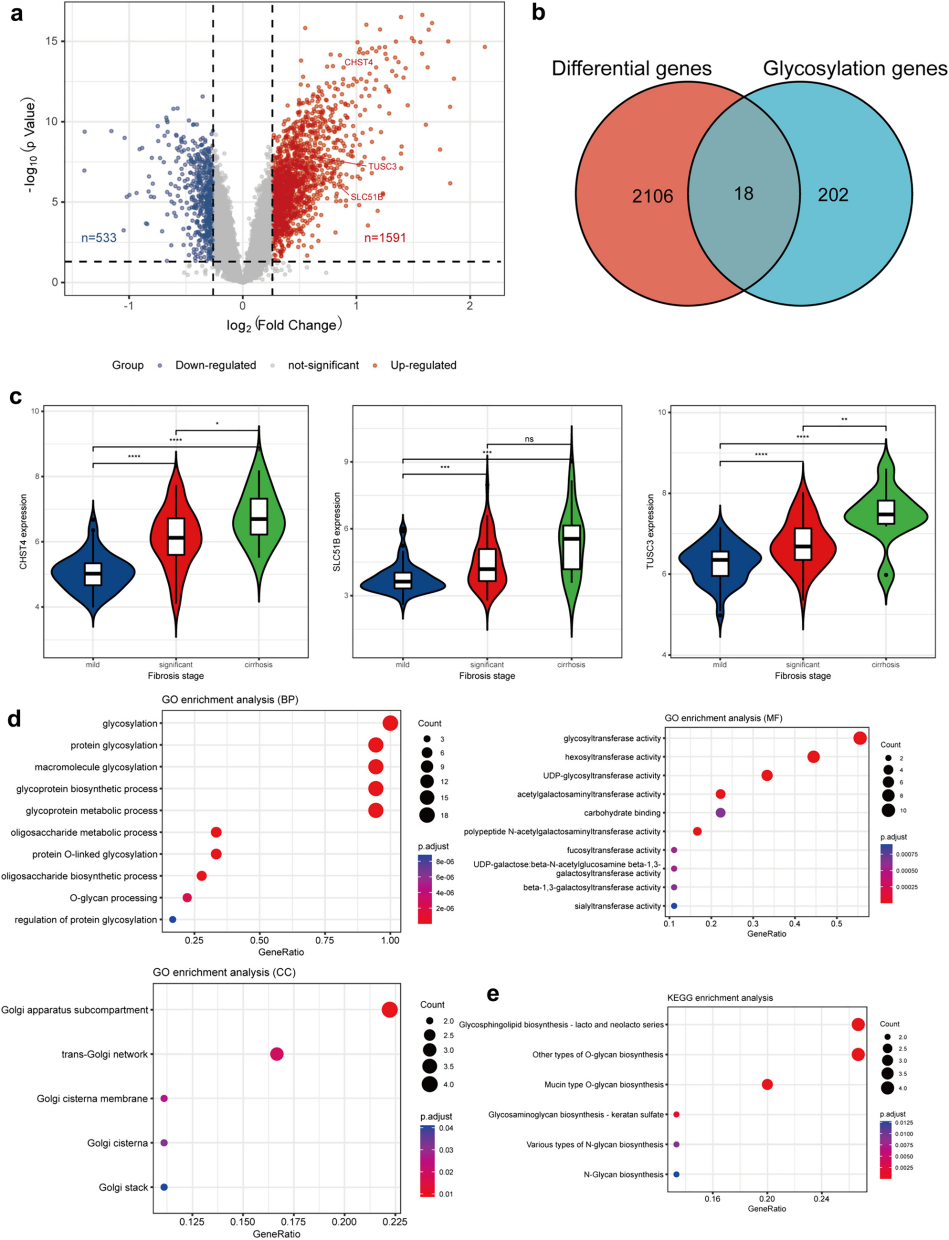
Relationship between glycosyltransferases and liver fibrosis. **a** Volcano plot of genes differentially expressed between significant fibrosis and mild fibrosis in the GSE84044 dataset. Blue nodes represent down-regulation in significant fibrosis; red nodes represent up-regulation; and gray nodes represent no significant difference between them. **b** Intersection of differentially expressed genes (DEGs) in the GSE84044 and glycosyltransferases-related genes. **c** Expression of 3 representative glycosyltransferase genes (CHST4, SLC51B, and TUSC3) in different fibrosis stage; **d** GO enrichment analysis of glycosyltransferases-related DEGs; **e** KEGG enrichment analysis of glycosyltransferases-related DEGs

In total, 2124 DEGs were identified by screening, of which 533 were downregulated (Fig. 5A, blue dot) and 1691 were upregulated genes (Fig. 5A, red dot). Then, we extracted glycosyltransferases-related genes from the “GSEA” database, as shown in Fig. 5B; the count on the left (2106 genes) refers to DEGs unique to GSE84044; the count in the middle (18 genes) refers to glycosyltransferases-related DEGs; and the count on the right (202 genes) refers to unique glycosyltransferases genes. These 18 glycosyltransferases-related DEGs were: B3GALNT1, B3GALT2, CHST4, EOGT, FUOM, FUT4, FUT8, GALNT10, GALNT12, GALNT7, IL15, PMM1, RAMP1, SLC51B, ST3GAL6, ST8SIA4, TMEM165, and TUSC3. Figure 5C shows the expression of three representative glycosyltransferase genes in different fibrosis stages. As fibrosis increased, the expression level of CHST4 (5.05 ± 0.55 vs. 6.12 ± 0.89 vs. 6.76 ± 0.79, *p* < 0.001) increased sequentially. SLC51B (3.76 ± 0.64 vs. 4.47 ± 1.09 vs. 5.34 ± 1.43, *p* < 0.001) and TUSC3 (6.27 ± 0.46 vs. 6.75 ± 0.59 vs. 7.47 ± 0.68, *p* < 0.001) were also similar.

Finally, we carried out GO and KEGG enrichment analysis on glycosyltransferases-related DEGs and found that glycosylation and glycosyltransferase-related pathways were significantly enriched (Fig. 5D, E). In summary, this implied that glycosylation related pathways were significantly activated in liver fibrosis. This provides us with a reference for a deeper understanding of the value of serum *N*-glycan in liver fibrosis.

## Discussion

This study comprehensively and definitively expounded the association between serum *N*-glycan markers, a novel biomarker, and liver fibrosis in CHB patients based on liver biopsy. Firstly, we found that serum *N*-glycan profiles P1 (NGA2F), P2 (NGA2FB), P4 (NG1A2F), P5 (NA2), P7 (NA2FB), P8 (NA3), and P11 (NA4Fb) were positively correlated with the stage of fibrosis, while P5 (NA2), P8 (NA3), and P10 (NA4) were negatively correlated with the stage of fibrosis. Then, we used LASSO regression to fit a Px score function for the diagnosis of liver fibrosis. We found that for per Px score increase, the risk of significant fibrosis was increased by 3.54 times; compared with the Px score bottom tertile, in patients in the top tertile, significant fibrosis risk increased by 3.58 times. Next, we compared the Px score with other non-invasive fibrosis markers (LSM, APRI, FIB-4) and found that the Px score was superior to others. Finally, the transcriptomic analysis revealed that glycosyl-transferase-related genes were positive correlated with liver fibrosis; enrichment analysis also indicated glycosylation and glycosyltransferase related pathways were significantly enriched.

The glycosylation modification process was ubiquitous. Besides liver fibrosis mentioned in this study, serum *N*-glycan profiles play a prominent vale in early diagnosis and monitoring of HCC. Zhuang et al. reported the results of early diagnosis of HCC using serum *N*-glycan (3397 cases enrolled, including 767 cases of liver cancer. not yet published) at the 12th National Conference of the CNSLD. The sensitivity and specificity of serum *N*-glycan in HCC diagnosis reached 86.44 and 90.04%. In hepatitis B-related liver disease individuals, the sensitivity was consistent with the total samples, the specificity was 93.69%. The sensitivity (86.1 vs. 49.4%) and specificity (93.9 vs. 87.0%) of serum *N*-glycan for detecting HCC were significantly higher than those of AFP (cutoff 20 ng/mL) [19]. Butaye et al. [14] systematic review, elucidated the role of different glycoproteins (whole serum, haptoglobin and vitronectin, glycosylated AFP and fucosylated kininogen, α-1-antitrypsin, and Golgi protein 73, and other glycoproteins) in the diagnosis of HCC. Guo et al. [20] study showed that NA2FB was abundant in patients with cirrhosis, while NA3Fb was abundant in HCC. The AUROC of NA3Fb (0.81 ± 0.07) and NA3Fb/NA2FB (0.87 ± 0.06) were superior to AFP (0.72 ± 0.09). NA3Fb/NA2FB combined with AFP had the best accuracy (AUROC: 0.89 ± 0.06) in the diagnosis of HCC. In addition, there has been progress in *N*-glycan biomarkers detection. Recently, Wang et al. developed a novel three-dimensional hierarchical porous carbon probe for the discovery of *N*-glycan biomarkers. The AUROC to distinguish healthy and liver diseases (hepatic dysfunction or HCC) was 0.95, and the AUROC to discern hepatic dysfunction and HCC was 0.85 [21]. Our previous study has shown significant differences in the expression of glycosyltransferase mRNA and protein in liver tissue of HCC (in 34 patients). The mRNA and protein expression levels of FUT8 and GnT-V genes in cancer tissues were significantly higher than in adjacent tissues. The mRNA expression level of the GnT-IVa gene in cancer was significantly higher than in adjacent tissues, while there was no significant difference in protein expression. These changes were consistent with the abundance of *N*-glycans in serum [22].

Non-invasive diagnosis of liver fibrosis was gradually superseding liver biopsy. Currently, both Chinese and international guidelines recommend LSM as a commonly used indicator for diagnosing liver fibrosis [2, 3, 23]. Several studies have shown its value in liver fibrosis diagnosis and follow-up. But there were also studies indicating its shortcomings. Our previous study (182 CHB patients receiving entecavir-based therapy were prospectively followed for 78 weeks for a second LSM and liver biopsy.) showed that a declining in liver stiffness cannot indicate fibrosis regression, but rather relieving of inflammation [24]. Ji et al. study (with 727 CHB patients) indicated that, after adjusting for confounding factors, changes in LSM (decrease ≥30%) were unreliable in estimating regression of fibrosis during treatment, which also supported our conclusion [25]. Although APRI and FIB-4 were widely used in the diagnosis and follow-up of viral hepatitis, they were developed based on hepatitis C [26]. Itakura et al. study, which included 1029 cases of hepatitis C and 384 cases of hepatitis B, showed that in chronic hepatitis C (CHC), APRI and FIB-4 increased significantly according to the degree of fibrosis (all *p* < 0.01). However, in CHB patients, APRI showed a slight increase without significance (*p* = 0.41). The AUROC of APRI and FIB-4 for diagnosis of advanced fibrosis was 0.781 and 0.796. On the other hand, the AUROCs were relatively lower in CHB cases compared with CHC (0.651 and 0.752, respectively) [27]. Aberra et al. study also suggested that using APRI as a non-invasive fibrosis indicator may result in failing to detect half of the patients in need of treatment (1190 Ethiopian CHB patients). APRI (at the WHO recommended threshold of 2.0) failed to identify most patients in need of treatment, with a sensitivity of 8.5% and a specificity of 99.3% [28]. In short, we still needed to further explore new non-invasive fibrosis indicators for the diagnosis and post-treatment monitoring of liver fibrosis. Serum *N*-glycan markers, as an emerging indicator, had many advantages in the early diagnosis of liver fibrosis and HCC, such as less quantity sample (only 20 µl of peripheral blood), high accuracy, and automated operation. It was worth further exploring.

There were also several inevitable limitations in our study. Firstly, owing to ethnic and HBV genotypes (mainly type B and C in China [29]) differences, further validation was needed for other ethnic groups. Wang et al. study has indicated that N/O-glycopatterns in human colostrum from different ethnic groups (Han, Hui, and Tibetan populations) in Northwest China were diverse [30]. Secondly, previous studies have shown that serum *N*-glycan markers played notable values in the diagnosis of HCC [14]. Unfortunately, this study was only a diagnostic cross-sectional study and did not follow up on the incidence of HCC in patients (especially cirrhosis patients). If these patients can detect the changes of serum *N*-glycan markers before imaging diagnosing HCC, it would be more conducive to the value of serum *N*-glycan markers in the following of CHB patients.

In summary, serum *N*-glycan markers were positively correlated with liver fibrosis. The Px score model had well performance in distinguishing significant fibrosis, and its diagnostic value was superior to commonly used non-invasive indicators (LSM, APRI, and FIB-4). Future studies were needed to investigate the effect of the use of the Px score and document cost-effectiveness of screening, which might eventually help reduce the large burden of CHB in the world.

## Supplementary Information

Below is the link to the electronic supplementary material.

Supplementary file1 (DOCX 340 KB)

## Data Availability

The raw data supporting the conclusions of this article will be made available by the authors upon reasonable request.

## Abbreviations

CHB: Chronic hepatitis B
DSA-FACE: DNA sequencer-assisted fluorophore-assisted with capillary electrophoresis
HCC: Hepatocellular carcinoma
LSM: Liver stiffness measurements
BMI: Body mass index
WBC: White blood cell
HGB: Hemoglobin
PLT: Platelet
ALT: Alanine aminotransferase
AST: Aspartate aminotransferase
ALP: Alkaline phosphatase
GGT: Glutamyl transpeptidase
ALB: Albumin
TBIL: Total bilirubin
DBIL: Direct bilirubin
TG: Total glyceride
TC: Total cholesterol
HDL: High-density lipoprotein
LDL: Low-density lipoprotein
AFP: Alpha-fetoprotein
PTA: Prothrombin activity
HE: Hematoxylin-eosin
GO: Gene Ontology
KEGG: Kyoto Encyclopedia of Genes and Genomes
SD: Standard deviation
IQR: Interquartile range
LASSO: Least absolute shrinkage and selection operator
GLM: Generalized linear model
ROC: Receiver operating characteristic
