# Supplementary material for: Dose–response relationship between serum N-glycan markers and liver fibrosis in chronic hepatitis B
Source: Hepatol Int. 2024 Jul 17;18(5):1434–47. doi: 10.1007/s12072-024-10709-y (PMC11461603; doi:10.1007/s12072-024-10709-y)
Supplement: Supplementary file 1 — Supplementary file1 (DOCX 340 KB) [file 12072_2024_10709_MOESM1_ESM.docx]

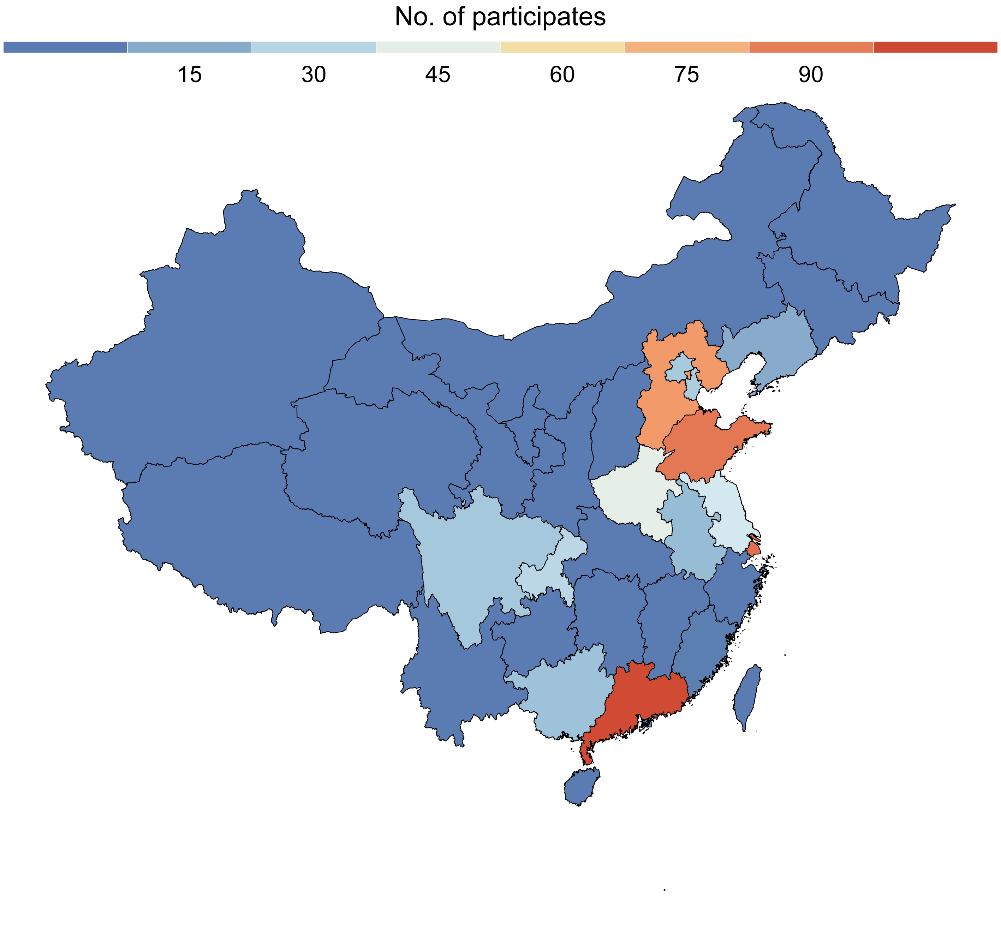


**Figure S1 Flow of participates included in the analysis**


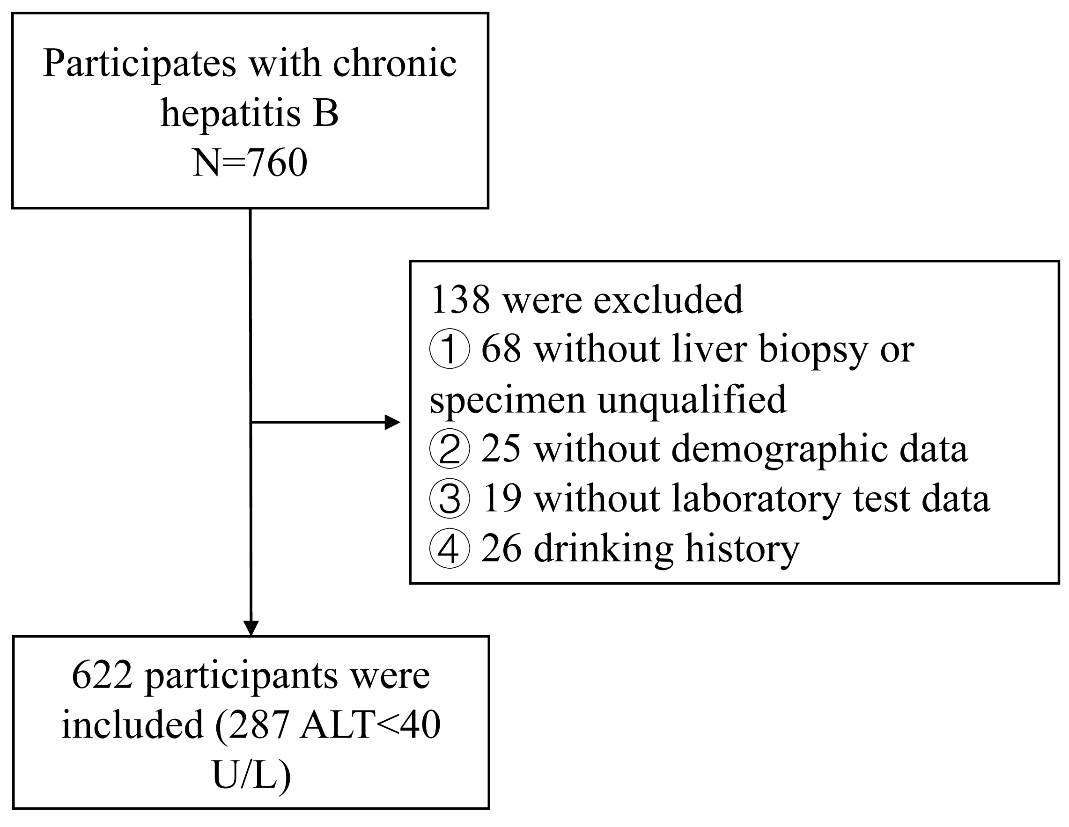


**Figure S2 Distribution of participants**

**Table S1 Baseline characteristics of all participants in different liver fibrosis stages**

|  | F 0 to 2 | F 3 to 4 | F 5 to 6 | P-value |
| --- | --- | --- | --- | --- |
| No. | 168 | 311 | 143 |  |
| Age (year) | 40.7 (9.9) 41.0 (33.8-47.0) | 41.9 (10.4) 42.0 (33.0-50.0) | 44.5 (9.6) 46.0 (37.5-52.0) | 0.003 |
| Sex |  |  |  | 0.021 |
| female | 62 (36.9%) | 95 (30.5%) | 32 (22.4%) |  |
| male | 106 (63.1%) | 216 (69.5%) | 111 (77.6%) |  |
| BMI (kg/m2) | 23.3 (3.0) 23.0 (21.2-25.1) | 23.8 (3.4) 23.6 (21.4-25.8) | 24.8 (3.5) 24.6 (22.2-27.4) | <0.001 |
| WBC (/10^9) | 5.4 (1.3) 5.2 (4.5-6.2) | 5.5 (1.6) 5.3 (4.4-6.2) | 5.3 (1.7) 5.2 (4.1-6.3) | 0.577 |
| HGB (g/L) | 144.5 (16.9) 145.0 (134.8-155.2) | 145.2 (17.2) 148.0 (136.0-159.0) | 146.1 (16.5) 149.0 (136.0-158.0) | 0.332 |
| PLT (/10^9) | 194.8 (54.2) 192.0 (162.0-223.2) | 171.7 (52.4) 169.0 (132.0-206.5) | 145.3 (47.7) 138.0 (109.0-173.0) | <0.001 |
| ALT (U/L) | 54.8 (73.2) 31.5 (23.6-50.5) | 103.5 (181.2) 45.0 (30.0-90.0) | 79.1 (108.3) 46.0 (34.5-71.5) | <0.001 |
| AST (U/L) | 37.4 (36.0) 27.8 (21.0-37.0) | 70.7 (129.3) 35.0 (26.1-60.0) | 71.6 (152.3) 37.0 (29.0-54.0) | <0.001 |
| ALP (U/L) | 75.8 (20.6) 76.0 (63.8-87.0) | 84.5 (30.5) 80.0 (63.0-99.0) | 96.2 (41.1) 89.0 (71.0-114.0) | <0.001 |
| GGT (U/L) | 33.0 (32.6) 22.0 (15.8-38.0) | 55.2 (61.7) 33.0 (21.0-65.0) | 77.5 (92.4) 52.0 (31.0-100.0) | <0.001 |
| ALB (g/L) | 44.8 (3.9) 45.0 (42.6-47.0) | 42.4 (4.5) 42.3 (39.3-45.5) | 41.9 (4.7) 42.3 (39.0-45.8) | <0.001 |
| TBIL (μmol/L) | 15.1 (6.5) 13.7 (10.7-18.4) | 17.5 (15.6) 15.0 (11.1-20.0) | 18.9 (11.2) 15.8 (12.2-21.2) | 0.004 |
| DBIL (μmol/L) | 4.2 (2.5) 3.7 (2.7-5.3) | 5.9 (8.9) 4.6 (3.2-6.6) | 6.3 (5.6) 4.7 (3.5-7.0) | <0.001 |
| TCHO (mmol/L) | 4.6 (0.9) 4.6 (4.1-5.2) | 4.3 (0.9) 4.3 (3.7-4.8) | 4.5 (0.9) 4.3 (3.9-5.0) | <0.001 |
| TG (mmol/L) | 1.2 (0.6) 1.0 (0.8-1.4) | 1.1 (0.7) 0.9 (0.8-1.3) | 1.2 (0.7) 1.1 (0.8-1.4) | 0.134 |
| HDL (mmol/L) | 1.4 (0.4) 1.4 (1.1-1.6) | 1.3 (0.3) 1.3 (1.1-1.6) | 1.3 (0.3) 1.3 (1.1-1.5) | 0.015 |
| LDL (mmol/L) | 2.8 (0.7) 2.7 (2.2-3.2) | 2.5 (0.8) 2.5 (1.9-3.0) | 2.7 (0.8) 2.6 (2.2-3.2) | 0.001 |
| AFP (ng/mL) | 8.2 (32.9) 2.9 (1.9-4.5) | 15.3 (39.1) 4.2 (2.3-9.9) | 22.5 (68.2) 4.9 (3.4-11.1) | <0.001 |
| PTA (%) | 99.2 (12.2) 100.0 (93.0-102.9) | 91.0 (12.2) 92.0 (83.3-100.0) | 86.2 (14.0) 87.0 (77.0-97.0) | <0.001 |
| LSM (kPa) | 7.2 (3.7) 6.1 (4.8-8.8) | 12.5 (8.3) 10.4 (7.6-14.6) | 17.8 (10.9) 15.5 (10.3-21.9) | <0.001 |
| CAP (dB/m) | 222.7 (47.0) 219.0 (198.0-249.0) | 217.3 (47.8) 216.0 (189.0-248.0) | 219.8 (52.4) 218.0 (188.0-249.0) | 0.722 |
| APRI | 0.5 (0.6) 0.4 (0.3-0.5) | 1.2 (2.3) 0.6 (0.4-1.0) | 1.5 (4.0) 0.7 (0.5-1.1) | <0.001 |
| FIB-4 | 1.2 (0.7) 1.0 (0.8-1.5) | 1.8 (1.6) 1.4 (0.9-2.2) | 2.5 (2.5) 1.8 (1.3-2.6) | <0.001 |
| HBV DNA (lg IU/mL) | 5.0 (2.3) 4.6 (3.3-7.1) | 5.4 (2.0) 5.4 (3.9-6.9) | 5.2 (1.8) 5.2 (3.9-6.4) | 0.058 |
| HBsAg (lg IU/mL) | 3.2 (1.0) 3.3 (2.7-3.8) | 3.3 (0.8) 3.3 (3.0-3.7) | 3.2 (0.7) 3.3 (3.0-3.5) | 0.804 |
| HBeAg |  |  |  | 0.005 |
| negative | 112 (67.1%) | 161 (51.9%) | 78 (54.5%) |  |
| positive | 55 (32.9%) | 149 (48.1%) | 65 (45.5%) |  |
| HBeAb |  |  |  | 0.653 |
| negative | 53 (31.7%) | 102 (33.0%) | 41 (28.7%) |  |
| positive | 114 (68.3%) | 207 (67.0%) | 102 (71.3%) |  |
| qAnti-HBc (lg IU/mL) | 3.9 (0.9) 4.1 (3.5-4.5) | 4.0 (0.8) 4.1 (3.6-4.5) | 4.0 (0.7) 4.1 (3.5-4.5) | 0.918 |
| HBV family history |  |  |  | 0.185 |
| no | 83 (49.4%) | 174 (55.9%) | 85 (59.4%) |  |
| yes | 85 (50.6%) | 137 (44.1%) | 58 (40.6%) |  |
| HCC family history |  |  |  | 0.127 |
| no | 140 (83.3%) | 279 (89.7%) | 126 (88.1%) |  |
| yes | 28 (16.7%) | 32 (10.3%) | 17 (11.9%) |  |
| HAI (Ishak) | 3.7 (2.2) 3.0 (2.0-4.0) | 5.9 (2.9) 5.0 (4.0-7.5) | 6.0 (2.6) 6.0 (4.0-7.0) | <0.001 |
| 0~4 | 130 (77.4%) | 108 (34.7%) | 43 (30.1%) |  |
| 5~6 | 23 (13.7%) | 95 (30.5%) | 49 (34.3%) |  |
| 7~9 | 11 (6.5%) | 71 (22.8%) | 37 (25.9%) |  |
| 10~18 | 4 (2.4%) | 37 (11.9%) | 14 (9.8%) |  |
| Splenomegaly |  |  |  | <0.001 |
| no | 126 (88.7%) | 209 (74.4%) | 68 (54.8%) |  |
| yes | 16 (11.3%) | 72 (25.6%) | 56 (45.2%) |  |

Data presented as mean (standard deviation) and median (quartile), while Gaussian distribution, compared with Student's t-test; Skewed distribution, compared with Kruskal-Wallis analysis) for continuous variables; number (percentage) for categorical variables (Chi-square or Fisher's exact tests). Fibrosis was measured by Ishak scoring system.

Abbreviations: BMI, body mass index; WBC, white blood cell; HGB, hemoglobin; PLT, platelet; ALT, alanine aminotransferase; AST, aspartate aminotransferase; ALP, alkaline phosphatase; GGT, glutamyl transpeptidase; ALB, albumin; TBIL, total bilirubin; DBIL, direct bilirubin; TG, total glyceride; TC, total cholesterol; HDL, high density lipoprotein; LDL, low density lipoprotein; AFP, alpha fetoprotein; PTA, prothrombin activity; HAI, histology activity index by Ishak fibrosis score; LSM, liver stiffness measurement; APRI, AST-to-platelet ratio index, [(AST/ULN)*100/PLT]; FIB-4, fibrosis index based on four factors, {(age*AST)/[PLT*(ALT^0.5)]}.

**Table S2 Serum *N*-glycan peaks in different fibrosis stage**

|  | F 0 to 2 | F 3 to 4 | F 5 to 6 | P-value |
| --- | --- | --- | --- | --- |
| No. | 168 | 311 | 143 |  |
| P0 | 1.28 (0.28) 1.23 (1.06-1.47) | 1.32 (0.34) 1.24 (1.11-1.46) | 1.36 (0.43) 1.26 (1.13-1.47) | 0.211 |
| P1 | 4.35 (1.71) 4.04 (3.27-5.12) | 5.60 (2.24) 5.14 (4.05-6.79) | 6.13 (2.66) 5.50 (4.06-7.63) | <0.001 |
| P2 | 0.73 (0.29) 0.70 (0.55-0.91) | 0.93 (0.40) 0.86 (0.67-1.15) | 1.09 (0.46) 0.98 (0.76-1.40) | <0.001 |
| P3 | 4.07 (0.96) 4.03 (3.45-4.62) | 4.64 (1.17) 4.46 (3.83-5.45) | 4.92 (1.30) 4.82 (3.93-5.72) | <0.001 |
| P4 | 2.99 (0.69) 2.92 (2.53-3.42) | 3.39 (0.80) 3.36 (2.83-3.85) | 3.54 (0.84) 3.42 (3.00-4.12) | <0.001 |
| P5 | 52.13 (3.96) 51.77 (49.82-54.44) | 49.46 (5.14) 49.88 (46.20-52.98) | 48.05 (5.82) 48.85 (44.14-52.41) | <0.001 |
| P6 | 15.62 (2.54) 15.50 (13.94-17.27) | 16.42 (2.95) 16.44 (14.57-18.09) | 16.64 (2.96) 16.40 (14.40-18.76) | 0.003 |
| P7 | 4.36 (1.17) 4.18 (3.57-5.00) | 4.95 (1.34) 4.77 (4.14-5.60) | 5.35 (2.05) 4.93 (4.25-6.17) | <0.001 |
| P8 | 8.89 (2.12) 8.88 (7.51-9.82) | 7.35 (1.89) 7.27 (6.15-8.62) | 6.83 (2.23) 6.56 (5.31-8.20) | <0.001 |
| P9 | 3.01 (1.48) 2.85 (1.92-4.05) | 3.57 (1.50) 3.58 (2.43-4.57) | 3.79 (1.48) 3.86 (2.87-4.81) | <0.001 |
| P10 | 1.97 (0.55) 1.93 (1.59-2.29) | 1.68 (0.51) 1.65 (1.33-2.00) | 1.58 (0.59) 1.51 (1.17-1.95) | <0.001 |
| P11 | 0.60 (0.24) 0.60 (0.44-0.76) | 0.69 (0.22) 0.69 (0.55-0.81) | 0.72 (0.24) 0.72 (0.60-0.88) | <0.001 |

**Table S3 Baseline characteristics of all participants according to Px tertile.**

|  | Bottom tertile (1.73-3.18) | Middle tertile (3.18-3.63) | Top tertile (3.64-5.01) | P-value |
| --- | --- | --- | --- | --- |
| No. | 207 | 207 | 208 |  |
| Age (year) | 38.7 (10.1) 39.0 (31.5-45.0) | 42.7 (9.7) 43.0 (34.5-50.0) | 45.0 (9.7) 46.0 (38.0-53.0) | <0.001 |
| Sex |  |  |  | <0.001 |
| female | 83 (40.1%) | 58 (28.0%) | 48 (23.1%) |  |
| male | 124 (59.9%) | 149 (72.0%) | 160 (76.9%) |  |
| BMI (kg/m2) | 23.7 (3.5) 23.4 (21.5-25.7) | 24.0 (3.5) 23.7 (21.5-26.0) | 24.0 (3.2) 24.0 (22.0-25.9) | 0.389 |
| WBC (/10^9) | 5.6 (1.5) 5.4 (4.5-6.4) | 5.4 (1.5) 5.2 (4.5-6.1) | 5.2 (1.6) 4.9 (4.1-6.1) | 0.012 |
| HGB (g/L) | 143.9 (18.1) 147.0 (133.5-156.0) | 146.3 (16.1) 149.0 (137.0-159.0) | 145.5 (16.6) 147.0 (135.0-159.0) | 0.321 |
| PLT (/10^9) | 198.7 (54.6) 196.0 (164.5-228.5) | 167.4 (50.0) 166.0 (132.0-198.5) | 149.6 (47.5) 144.0 (111.0-182.2) | <0.001 |
| ALT (U/L) | 51.1 (65.8) 32.0 (22.0-54.0) | 80.1 (120.8) 41.0 (29.0-72.3) | 122.9 (203.4) 56.0 (37.0-106.2) | <0.001 |
| AST (U/L) | 35.3 (30.5) 27.0 (21.9-36.0) | 54.2 (73.9) 33.0 (25.0-48.9) | 96.0 (185.5) 43.0 (32.6-73.0) | <0.001 |
| ALP (U/L) | 74.8 (21.4) 75.0 (59.0-85.0) | 83.5 (25.6) 79.0 (66.0-100.0) | 96.1 (41.3) 87.0 (71.0-110.0) | <0.001 |
| GGT (U/L) | 34.0 (32.2) 23.0 (16.0-39.8) | 52.5 (52.1) 31.0 (20.5-65.0) | 76.1 (92.1) 49.0 (29.0-90.5) | <0.001 |
| ALB (g/L) | 44.6 (4.2) 45.0 (42.0-47.3) | 43.2 (4.1) 43.6 (40.4-46.1) | 41.1 (4.6) 41.2 (38.2-44.4) | <0.001 |
| TBIL (μmol/L) | 15.6 (7.2) 13.9 (10.6-19.1) | 15.7 (7.4) 14.1 (10.8-19.0) | 20.2 (19.3) 16.6 (12.5-21.9) | <0.001 |
| DBIL (μmol/L) | 4.4 (2.6) 3.9 (2.8-5.5) | 4.9 (3.3) 4.2 (2.9-5.7) | 7.3 (11.1) 5.2 (3.6-7.6) | <0.001 |
| TCHO (mmol/L) | 4.5 (0.9) 4.5 (4.0-5.0) | 4.5 (0.9) 4.4 (3.9-5.0) | 4.3 (0.9) 4.2 (3.7-4.9) | 0.043 |
| TG (mmol/L) | 1.2 (0.7) 1.0 (0.8-1.5) | 1.3 (0.8) 1.0 (0.8-1.4) | 1.1 (0.5) 0.9 (0.8-1.2) | 0.153 |
| HDL (mmol/L) | 1.3 (0.3) 1.3 (1.1-1.5) | 1.4 (0.4) 1.3 (1.1-1.6) | 1.4 (0.3) 1.3 (1.1-1.6) | 0.394 |
| LDL (mmol/L) | 2.7 (0.8) 2.6 (2.1-3.2) | 2.6 (0.8) 2.6 (2.1-3.0) | 2.5 (0.8) 2.5 (2.0-3.0) | 0.04 |
| AFP (ng/mL) | 6.6 (21.6) 2.9 (1.9-5.0) | 10.9 (36.2) 3.9 (2.3-6.5) | 27.4 (66.4) 6.7 (3.3-15.6) | <0.001 |
| PTA (%) | 96.6 (13.5) 98.3 (88.0-102.9) | 92.9 (12.3) 93.4 (85.0-100.0) | 86.8 (12.7) 88.0 (77.0-99.2) | <0.001 |
| LSM (kPa) | 8.6 (4.9) 7.2 (5.0-10.8) | 11.2 (7.0) 9.5 (6.2-13.2) | 17.1 (11.3) 14.0 (9.2-21.3) | <0.001 |
| CAP (dB/m) | 219.9 (45.2) 220.5 (195.0-246.8) | 224.1 (55.0) 222.0 (193.0-257.8) | 214.0 (44.9) 212.0 (187.0-241.0) | 0.107 |
| APRI | 0.5 (0.5) 0.3 (0.2-0.5) | 0.9 (1.5) 0.5 (0.4-0.9) | 1.9 (4.0) 0.8 (0.5-1.6) | <0.001 |
| FIB-4 | 1.1 (0.6) 0.9 (0.7-1.4) | 1.7 (1.3) 1.4 (1.0-2.0) | 2.6 (2.5) 2.0 (1.3-3.0) | <0.001 |
| HBV DNA (lg IU/mL) | 4.9 (2.2) 4.5 (3.2-6.7) | 5.1 (2.0) 4.9 (3.5-6.5) | 5.7 (1.9) 5.7 (4.3-7.2) | <0.001 |
| HBsAg (lg IU/mL) | 3.2 (1.0) 3.3 (2.8-3.7) | 3.2 (0.8) 3.3 (2.9-3.6) | 3.3 (0.7) 3.4 (2.9-3.6) | 0.938 |
| HBeAg |  |  |  | 0.03 |
| negative | 128 (62.1%) | 120 (58.3%) | 103 (49.5%) |  |
| positive | 78 (37.9%) | 86 (41.7%) | 105 (50.5%) |  |
| HBeAb |  |  |  | 0.865 |
| negative | 62 (30.2%) | 67 (32.5%) | 67 (32.2%) |  |
| positive | 143 (69.8%) | 139 (67.5%) | 141 (67.8%) |  |
| qAnti-HBc (lg IU/mL) | 3.8 (0.8) 4.1 (3.5-4.4) | 3.9 (0.8) 4.1 (3.5-4.5) | 4.1 (0.7) 4.2 (3.6-4.6) | 0.011 |
| HBV family history |  |  |  | 0.396 |
| no | 106 (51.2%) | 119 (57.5%) | 117 (56.2%) |  |
| yes | 101 (48.8%) | 88 (42.5%) | 91 (43.8%) |  |
| HCC family history |  |  |  | 0.259 |
| no | 176 (85.0%) | 187 (90.3%) | 182 (87.5%) |  |
| yes | 31 (15.0%) | 20 (9.7%) | 26 (12.5%) |  |
| HAI (Ishak) |  |  |  | <0.001 |
| 0~4 | 137 (66.2%) | 91 (44.0%) | 53 (25.5%) |  |
| 5~6 | 41 (19.8%) | 60 (29.0%) | 66 (31.7%) |  |
| 7~9 | 18 (8.7%) | 44 (21.3%) | 57 (27.4%) |  |
| 10~18 | 11 (5.3%) | 12 (5.8%) | 32 (15.4%) |  |
| Splenomegaly |  |  |  | <0.001 |
| no | 154 (83.7%) | 141 (76.2%) | 108 (60.7%) |  |
| yes | 30 (16.3%) | 44 (23.8%) | 70 (39.3%) |  |

Data presented as mean (standard deviation) and median (quartile), while Gaussian distribution, compared with Student's t-test; Skewed distribution, compared with Kruskal-Wallis analysis) for continuous variables; number (percentage) for categorical variables (Chi-square or Fisher's exact tests). Fibrosis was measured by Ishak scoring system.

Abbreviations: BMI, body mass index; WBC, white blood cell; HGB, hemoglobin; PLT, platelet; ALT, alanine aminotransferase; AST, aspartate aminotransferase; ALP, alkaline phosphatase; GGT, glutamyl transpeptidase; ALB, albumin; TBIL, total bilirubin; DBIL, direct bilirubin; TG, total glyceride; TC, total cholesterol; HDL, high density lipoprotein; LDL, low density lipoprotein; AFP, alpha fetoprotein; PTA, prothrombin activity; HAI, histology activity index by Ishak fibrosis score; LSM, liver stiffness measurement; APRI, AST-to-platelet ratio index, [(AST/ULN)*100/PLT]; FIB-4, fibrosis index based on four factors, {(age*AST)/[PLT*(ALT^0.5)]}.

**Table S4 Characteristics of patients in training and validation sets**

|  | Training | Validation | P-value |
| --- | --- | --- | --- |
| No. | 311 | 311 |  |
| Age (year) | 41.3 (10.5) 41.0 (33.0-48.0) | 43.0 (9.8) 42.0 (35.0-50.0) | 0.063 |
| Sex |  |  | 0.542 |
| female | 91 (29.3%) | 98 (31.5%) |  |
| male | 220 (70.7%) | 213 (68.5%) |  |
| BMI (kg/m^2^) | 23.8 (3.2) 23.7 (21.6-25.8) | 23.9 (3.6) 23.6 (21.6-25.9) | 0.883 |
| WBC (/10^9) | 5.4 (1.5) 5.2 (4.3-6.2) | 5.5 (1.6) 5.3 (4.4-6.2) | 0.351 |
| HGB (g/L) | 145.8 (17.0) 148.0 (135.5-159.0) | 144.7 (16.9) 147.0 (135.0-156.0) | 0.407 |
| PLT (/10^9) | 172.1 (57.2) 168.0 (132.0-209.5) | 171.6 (52.0) 169.0 (132.5-203.5) | 0.852 |
| ALT (U/L) | 96.7 (171.2) 43.0 (28.6-78.5) | 72.8 (111.2) 41.0 (26.8-69.0) | 0.35 |
| AST (U/L) | 68.4 (131.2) 33.0 (25.0-54.7) | 55.4 (105.9) 33.0 (25.0-48.0) | 0.522 |
| ALP (U/L) | 83.8 (31.3) 79.0 (63.0-99.0) | 85.8 (32.4) 80.0 (67.0-99.0) | 0.446 |
| GGT (U/L) | 56.0 (77.7) 34.0 (21.0-64.0) | 52.4 (52.0) 31.0 (19.0-63.0) | 0.37 |
| ALB (g/L) | 43.2 (4.4) 43.1 (40.1-46.2) | 42.7 (4.6) 43.0 (39.7-46.0) | 0.271 |
| TBIL (μmol/L) | 17.7 (15.2) 15.3 (11.4-20.2) | 16.7 (9.8) 14.6 (11.0-19.6) | 0.255 |
| DBIL (μmol/L) | 5.8 (8.5) 4.5 (3.2-6.6) | 5.3 (5.0) 4.2 (3.0-5.9) | 0.091 |
| TCHO (mmol/L) | 4.4 (0.9) 4.3 (3.8-4.8) | 4.5 (0.9) 4.4 (3.9-5.1) | 0.043 |
| TG (mmol/L) | 1.1 (0.6) 1.0 (0.8-1.3) | 1.2 (0.7) 1.0 (0.8-1.4) | 0.586 |
| HDL (mmol/L) | 1.3 (0.3) 1.3 (1.1-1.5) | 1.4 (0.4) 1.3 (1.1-1.6) | 0.186 |
| LDL (mmol/L) | 2.6 (0.8) 2.5 (2.0-3.0) | 2.7 (0.8) 2.6 (2.1-3.1) | 0.173 |
| AFP (ng/mL) | 12.6 (33.4) 3.9 (2.3-7.9) | 17.5 (56.4) 4.0 (2.3-8.6) | 0.86 |
| PTA (%) | 91.7 (13.1) 92.0 (83.7-100.0) | 92.5 (13.8) 94.1 (83.2-100.0) | 0.429 |
| LSM (kPa) | 12.0 (8.3) 9.8 (6.3-14.6) | 12.6 (9.5) 10.0 (6.3-15.1) | 0.683 |
| CAP (dB/m) | 220.5 (48.2) 220.0 (191.0-251.0) | 218.2 (49.1) 216.0 (192.2-244.0) | 0.396 |
| APRI | 1.2 (2.6) 0.6 (0.3-1.0) | 1.0 (2.6) 0.5 (0.3-0.9) | 0.405 |
| FIB-4 | 1.8 (1.8) 1.4 (0.9-2.1) | 1.8 (1.7) 1.3 (0.9-2.1) | 0.895 |
| HBV DNA (lg IU/mL) | 5.3 (2.1) 5.3 (3.6-6.9) | 5.2 (2.0) 5.2 (3.8-6.6) | 0.643 |
| HBsAg (lg IU/mL) | 3.3 (0.9) 3.4 (3.0-3.7) | 3.2 (0.8) 3.2 (2.9-3.6) | 0.035 |
| HBeAg |  |  | 0.224 |
| negative | 168 (54.2%) | 183 (59.0%) |  |
| positive | 142 (45.8%) | 127 (41.0%) |  |
| HBeAb |  |  | 0.403 |
| negative | 103 (33.2%) | 93 (30.1%) |  |
| positive | 207 (66.8%) | 216 (69.9%) |  |
| qAnti-HBc (lg IU/mL) | 3.9 (0.8) 4.1 (3.5-4.5) | 4.0 (0.7) 4.2 (3.6-4.5) | 0.178 |
| HBV family history |  |  | 0.42 |
| no | 166 (53.4%) | 176 (56.6%) |  |
| yes | 145 (46.6%) | 135 (43.4%) |  |
| HCC family history |  |  | 0.181 |
| no | 267 (85.9%) | 278 (89.4%) |  |
| yes | 44 (14.1%) | 33 (10.6%) |  |
| HAI (Ishak) |  |  | 0.942 |
| 0~4 | 140 (45.0%) | 141 (45.3%) |  |
| 5~6 | 81 (26.0%) | 86 (27.7%) |  |
| 7~9 | 61 (19.6%) | 58 (18.6%) |  |
| 10~18 | 29 (9.3%) | 26 (8.4%) |  |
| Splenomegaly |  |  | 0.947 |
| no | 200 (73.8%) | 203 (73.6%) |  |
| yes | 71 (26.2%) | 73 (26.4%) |  |
| F (Ishak) |  |  | 0.577 |
| 0~2 | 81 (26.0%) | 87 (28.0%) |  |
| 3~4 | 162 (52.1%) | 149 (47.9%) |  |
| 5~6 | 68 (21.9%) | 75 (24.1%) |  |

Data presented as mean (standard deviation) and median (quartile), while Gaussian distribution, compared with Student's t-test; Skewed distribution, compared with Kruskal-Wallis analysis) for continuous variables; number (percentage) for categorical variables (Chi-square or Fisher's exact tests). Fibrosis was measured by Ishak scoring system.

Abbreviations: BMI, body mass index; WBC, white blood cell; HGB, hemoglobin; PLT, platelet; ALT, alanine aminotransferase; AST, aspartate aminotransferase; ALP, alkaline phosphatase; GGT, glutamyl transpeptidase; ALB, albumin; TBIL, total bilirubin; DBIL, direct bilirubin; TG, total glyceride; TC, total cholesterol; HDL, high density lipoprotein; LDL, low density lipoprotein; AFP, alpha fetoprotein; PTA, prothrombin activity; HAI, histology activity index by Ishak fibrosis score; LSM, liver stiffness measurement; APRI, AST-to-platelet ratio index, [(AST/ULN)*100/PLT]; FIB-4, fibrosis index based on four factors, {(age*AST)/[PLT*(ALT^0.5)]}.

**Table S5 Serum *N*-glycan peaks in training and validation sets**

|  | Training | Validation | P-value |
| --- | --- | --- | --- |
| No. | 311 | 311 |  |
| P0 | 1.31 (0.33) 1.25 (1.10-1.44) | 1.33 (0.37) 1.25 (1.11-1.50) | 0.41 |
| P1 | 5.37 (2.38) 4.84 (3.58-6.80) | 5.39 (2.25) 4.94 (3.91-6.38) | 0.594 |
| P2 | 0.90 (0.41) 0.81 (0.61-1.13) | 0.93 (0.41) 0.86 (0.64-1.12) | 0.247 |
| P3 | 4.55 (1.19) 4.39 (3.76-5.37) | 4.55 (1.19) 4.41 (3.67-5.34) | 0.955 |
| P4 | 3.29 (0.83) 3.22 (2.71-3.75) | 3.35 (0.78) 3.30 (2.81-3.81) | 0.288 |
| P5 | 49.88 (5.32) 50.30 (46.53-53.53) | 49.83 (5.15) 50.29 (46.96-53.11) | 0.992 |
| P6 | 16.33 (2.96) 16.20 (14.25-17.92) | 16.17 (2.78) 16.08 (14.16-18.08) | 0.584 |
| P7 | 4.82 (1.30) 4.68 (4.00-5.49) | 4.94 (1.74) 4.71 (3.88-5.63) | 0.842 |
| P8 | 7.68 (2.16) 7.59 (6.16-9.04) | 7.61 (2.20) 7.52 (6.21-8.96) | 0.789 |
| P9 | 3.44 (1.48) 3.41 (2.38-4.54) | 3.50 (1.55) 3.52 (2.36-4.45) | 0.745 |
| P10 | 1.75 (0.55) 1.72 (1.34-2.08) | 1.72 (0.56) 1.68 (1.35-2.12) | 0.582 |
| P11 | 0.67 (0.23) 0.68 (0.52-0.82) | 0.67 (0.24) 0.68 (0.52-0.81) | 0.86 |

**Table S6 Comparison of the efficacy of serum *N*-glycan Px model, LSM, APRI, and FIB-4 in diagnosing significant fibrosis in training and validation sets**

|  | AUROC (95% CI) | Sensitivity | Specificity | PPV | NPV | PLR | NLR |
| --- | --- | --- | --- | --- | --- | --- | --- |
| Training set |  |  |  |  |  |  |  |
| Px | 0.754 (0.694-0.814) | 0.757 | 0.642 | 0.857 | 0.482 | 2.113 | 0.379 |
| LSM | 0.756 (0.7-0.812) | 0.809 | 0.704 | 0.886 | 0.564 | 2.729 | 0.272 |
| APRI | 0.743 (0.681-0.805) | 0.652 | 0.790 | 0.898 | 0.444 | 3.107 | 0.440 |
| FIB-4 | 0.712 (0.651-0.773) | 0.678 | 0.679 | 0.857 | 0.426 | 2.113 | 0.474 |
| Validation set |  |  |  |  |  |  |  |
| Px | 0.766 (0.711-0.822) | 0.638 | 0.816 | 0.899 | 0.467 | 3.471 | 0.443 |
| LSM | 0.675 (0.616-0.733) | 0.786 | 0.563 | 0.822 | 0.505 | 1.799 | 0.381 |
| APRI | 0.655 (0.589-0.72) | 0.746 | 0.506 | 0.795 | 0.436 | 1.508 | 0.503 |
| FIB-4 | 0.655 (0.589-0.72) | 0.746 | 0.506 | 0.795 | 0.436 | 1.508 | 0.503 |
